# Purulent pleurisy caused by Salmonella enterica subspecies arizonae: a case report

**DOI:** 10.1099/acmi.0.000985.v5

**Published:** 2025-06-20

**Authors:** Amine Amri, Youssra Boughalem, Elmostafa Benaissa, Yassine Benlahlou, Mariama Chadli

**Affiliations:** 1 Bacteriology Laboratory, Mohammed V Military Training Hospital, Rabat, Morocco; 2 Faculty of Medicine and Pharmacy, Rabat, Morocco; 3 Faculty of Medicine and Pharmacy, Marrakech, Morocco

**Keywords:** infection, pleurisy, *Salmonella*

## Abstract

**Background.** Salmonellosis most commonly presents clinically as typhoid fever or gastroenteritis. Pleuropulmonary infections due to *Salmonella* are still rare, even though they have often been described in immunocompromised patients.

**Case presentation.** We report a rare case of purulent pleurisy caused by *Salmonella enterica* subsp. *arizonae*, occurring in a 50-year-old female with breast cancer who is currently treated with chemotherapy and radiotherapy along with chronic renal failure requiring haemodialysis, who presented with acute chest pain, dyspnoea and haemodynamic instability. After bacteriological identification of *Salmonella enterica* subsp. *arizonae* in pleural fluid, antibiotic susceptibility testing was performed. The patient was then started on a broad-spectrum antibiotic, which successfully improved her condition.

**Conclusion.** Our case highlights the implication of *Salmonella enterica* subsp. *arizonae* in purulent pleurisy in an immunocompromised patient. An early diagnosis and a proper antibiotic therapy enabled us to reduce the morbidity and mortality risk in our patient.

## Data Summary

All data associated with this work is reported within the article.

## Introduction

*Salmonella* is a Gram-negative bacillus that belongs to the *Enterobacteriaceae* family [1]. It is widely responsible for various human infections, including human typhoidal and non-typhoidal salmonellosis [2]. Non-typhoidal *Salmonella* infections are usually transmitted through the ingestion of contaminated food or water or contact with reptiles and their habitat [3]. After ingestion, the bacteria colonize the small intestine and can cause gastrointestinal symptoms such as diarrhoea, abdominal pain and fever [4]. In some cases, it can spread beyond the intestine, leading to severe infections such as bacteraemia, urinary tract infections, endocarditis or joint infections [4]. Pleuropulmonary infections due to *Salmonella* are still rare even though they have often been described in immunocompromised patients [3,5]. *Salmonella enterica* subsp. *arizonae* is a rare subspecies of *Salmonella* primarily associated with reptiles, particularly snakes and lizards [1]. It can cause infections in humans, often through direct contact with infected reptiles or by ingesting contaminated water or food [1]. We report a rare case of purulent pleurisy caused by *Salmonella enterica* subsp. *arizonae* in an immunocompromised host.

## Case presentation

A 50-year-old female patient originally from Rabat, with a medical history of breast cancer currently treated with chemotherapy and radiotherapy and chronic renal failure requiring thrice-weekly haemodialysis, was admitted to the medical-surgical emergency department due to haemodynamic instability.

On physical examination, she presented with acute chest pain, dyspnoea, hypotension, bradycardia and a fever of 39 °C. When questioning the patient, she did not reveal any evidence of gastroenteritis in the days prior to the disorder. She also stated that she had not travelled abroad recently, was only eating well-cooked food and drinking bottled water due to her chemotherapy and radiotherapy sessions and had not been in contact with reptiles and amphibians. Thoracic angiography excluded the presence of a proximal pulmonary embolism but revealed a large pleural effusion associated with adjacent passive atelectasis. A pleural puncture allowed the drainage of a cloudy fluid that was then collected into two sterile vials for bacteriological and biochemical examinations. Samples for blood cultures were drawn and showed no growth within 5 days of incubation.

**Fig. 1.**
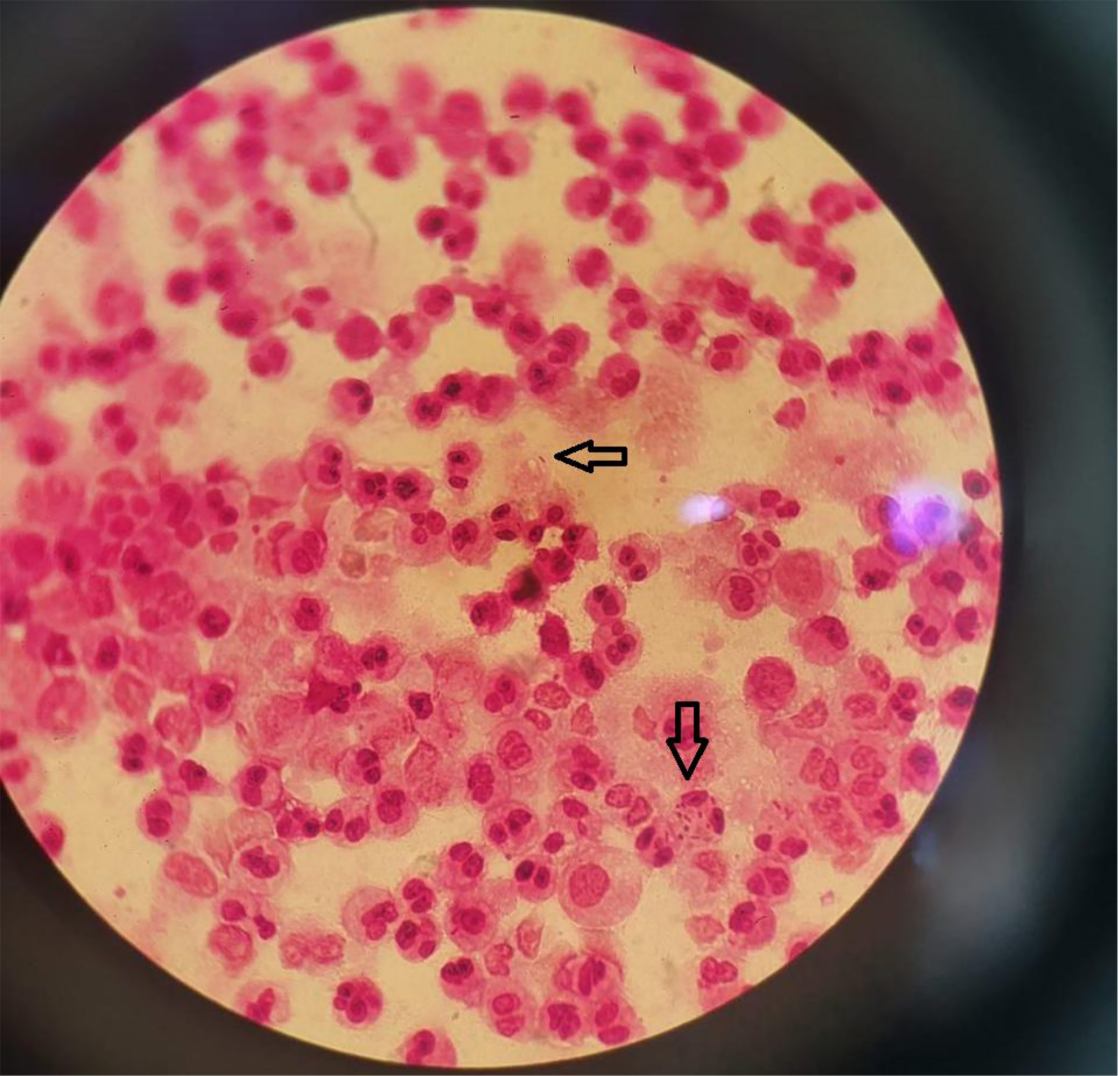
Pleural fluid Gram stain showing Gram-negative bacilli (arrow).

Blood cell count revealed leucocytosis at 12,000 mm^−^³, with neutrophil predominance, normochromic normocytic anaemia and thrombocytopenia at 27,000 mm^−^³. Infectious markers showed a C-reactive protein level of 236.4 mg l^−1^ and a procalcitonin level of 29.02 ng l^−1^. The rest of the blood tests showed no abnormalities. Microscopic examination of the pleural fluid revealed leucocytosis at 64,000 mm^−^³ with a predominance of neutrophils (87%), along with Gram-negative bacilli on the Gram stain (Fig. 1). Biochemical analysis of the pleural fluid was in favour of an exudate, with total proteins at 36 g l^−1^.

**Fig. 2.**
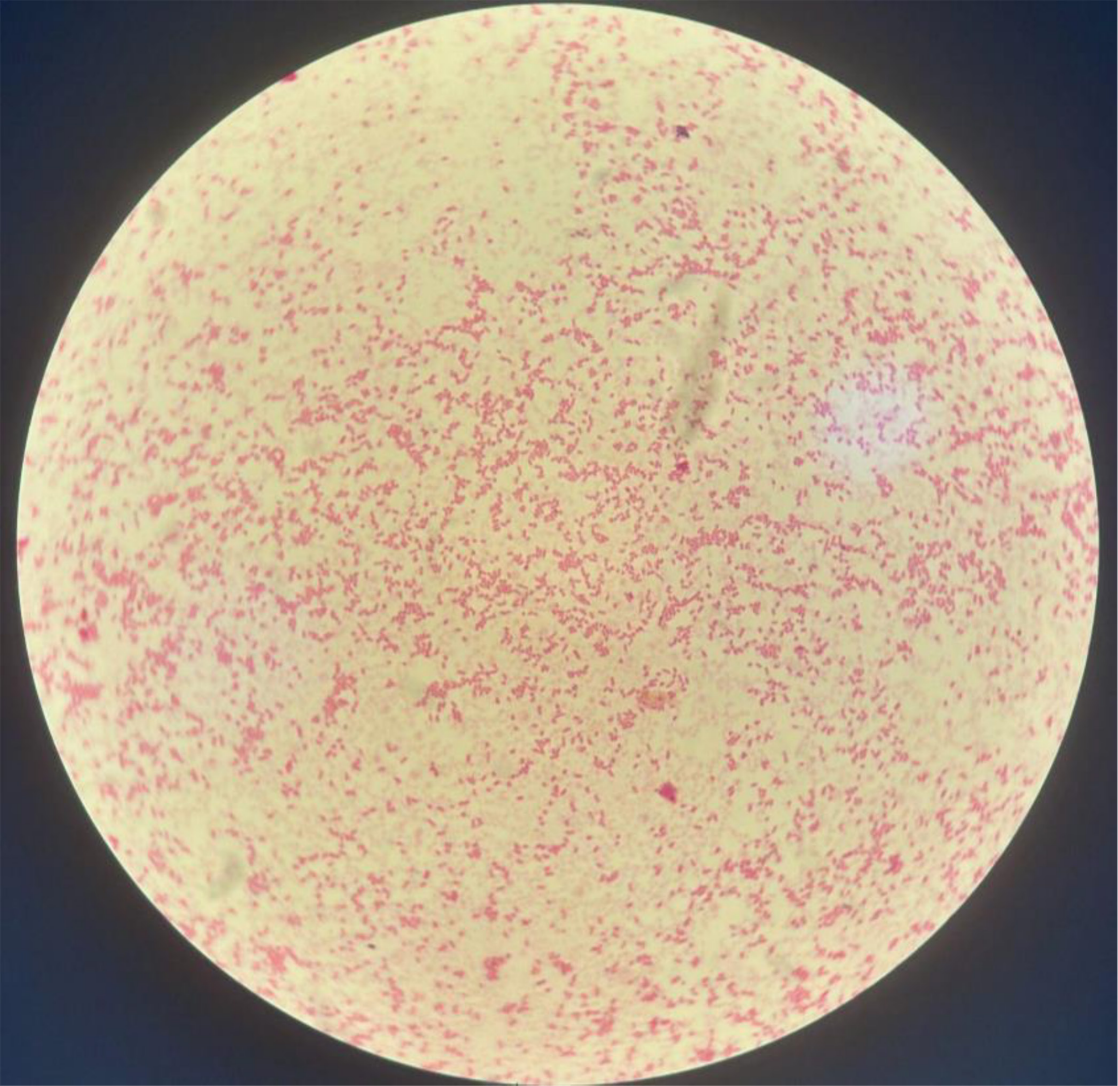
Gram staining showing regular Gram-negative bacilli under 1,000× magnification.

**Fig. 3.**
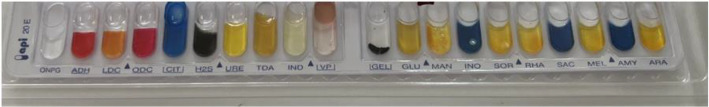
Biochemical and enzymatic characterization of *Salmonella enterica* subsp. *arizonae* carried out at the laboratory of the Mohammed V Military Hospital.

Bacterial culture, which included enrichment on blood culture vials, showed growth after 24 h of incubation. Microscopic examination of the culture isolates after Gram staining revealed regular Gram-negative bacilli (Fig. 2). The API 20E gallery (bioMérieux) allowed the identification of *Salmonella enterica* subsp. *arizonae* (99.7%) with the numerical profile: 7704552 (Fig. 3). Strain serotyping was unavailable to this date.

Antibiotic susceptibility testing was performed using the diffusion method on Mueller–Hinton agar and interpreted according to the 2024 European Committee on Antimicrobial Susceptibility Testing recommendations [6]. The antibiogram showed that our isolate was sensitive to amoxicillin, amoxicillin-clavulanic acid, cefoxitin, ceftriaxone, cefixime, cefepime, piperacillin-tazobactam, ertapenem, trimethoprim-sulfamethoxazole and levofloxacin (Table 1).

**Table 1.** Results of antimicrobial susceptibility testing

Antimicrobial agent	Disc content (µg)	Breakpoint (mm)		Diffusion diameters (mm)	Interpretation
		S ≥	R <		
**Amoxicillin**	20	19	19	**21**	Sensitive
**Amoxicillin-clavulanic acid**	20–10	19	19	**22**	Sensitive
**Piperacillin-tazobactam**	30–6	20	20	**24**	Sensitive
**Cefoxitin**	30	18	18	**20**	Sensitive
**Ceftriaxone**	30	25	22	**28**	Sensitive
**Cefixime**	5	17	17	**21**	Sensitive
**Cefepime**	30	27	24	**27**	Sensitive
**Ertapenem**	10	23	23	**25**	Sensitive
**Levofloxacin**	5	23	19	**25**	Sensitive
**Trimethoprim-sulfamethoxazole**	1.25–23.75	14	11	**20**	Sensitive

R, resistant; S, sensitive.

The patient was then started on ceftriaxone (1 g IV daily) and showed a good clinical and biological outcome within 7 days of treatment.

## Discussion

*Salmonella* is a Gram-negative, usually motile, facultative anaerobic bacillus that was first described in the 1880s by Urlapu and *et al*. [7]. *Salmonella* species are intracellular bacteria that were shown to have a tropism for abnormal tissue cells like malignant tumours, aneurysms and bone infarcts [7]. Underlying conditions like acquired immune deficiency syndrome (AIDS), malignancy, antineoplastic treatments, diabetes mellitus, chronic kidney disease, alcohol consumption, intravenous drug use, iron overload and long-term corticosteroid therapy compromise the patient’s immune system and, therefore, make them more susceptible to *Salmonella* purulent pleurisy [8]. Our patient showed various risk factors such as breast cancer, chemotherapy, radiotherapy and chronic renal failure which significantly increased her risk of developing the infection.

Pleuropulmonary infections can occur through direct spread from a nearby infected site, aspiration of gastric fluids in patients presenting with a gastrointestinal infection and dissemination via the bloodstream [1]. Although our diagnosis was challenged by the absence of gastrointestinal symptoms and bacteraemia, literature has shown that only 33% of the patients presenting with a non-typhoidal *Salmonella* infection have reported a history of gastrointestinal disease, and blood cultures are often negative due to a low bacterial load [9]. It is said that following an acute infection, *Salmonella* species can remain in a dormant state in the reticuloendothelial system, from where they can reactivate and disseminate haematogenously [9]. Effective clearance of the pathogen relies on cellular immunity [7].

Cases of *Salmonella enterica* subsp. *arizonae* purulent pleurisy are still rare. Only a few cases have been described [1,9,11]. A literature review of case reports of purulent pleurisy associated with *Salmonella enterica* subsp. *arizonae* has been listed in Table 2. *Salmonella enterica* subsp. *arizonae* is primarily associated with reptiles and is an unusual human pathogen [1]. In these studies (Table 2), all of the patients had underlying conditions that predisposed them to developing purulent pleurisy, and half of them did not report any known source of infection. This suggests that there might be additional, yet unidentified, sources that may cause the infection.

**Table 2.** Cases of purulent pleurisy caused by *Salmonella enterica* subsp. *arizonae*

Reference	Age	Gender	Underlying conditions	Source of infection	Outcome
[10]	25	Male	AIDS	Rattlesnake capsule	Improved
[10]	51	Male	Congestive heart failure	Rattlesnake capsule	Improved
[11]	38	Male	Gastric carcinoma	Rattlesnake capsule	Improved
[1]	72	Female	Systemic lupus erythematosus, colon cancer, malignant lymphoma	Unknown	Death
[9]	38	Male	Alcohol and methamphetamine misuse	Unknown	Improved
Present case	50	Female	Breast cancer, chemotherapy, radiotherapy, chronic renal failure	Unknown	Improved

The treatment of *Salmonella* infections varies depending on the severity of the infection, with antibiotics such as fluoroquinolones and third-generation cephalosporins used in severe cases [12]. In our case, the patient was put on a course of ceftriaxone, based on antimicrobial sensitivity testing results, which enabled her to be treated successfully.

## Conclusion

Our case highlights the implication of *Salmonella enterica* subsp. *arizonae* in purulent pleurisy in an immunocompromised patient. Although the original source of infection was not discovered, an early diagnosis and a proper antibiotic therapy enabled us to reduce the morbidity and mortality risk in our patient.

## Abbreviations

AIDS: acquired immune deficiency syndrome
